# Airways resistance and specific conductance for the diagnosis of obstructive airways diseases

**DOI:** 10.1186/s12931-015-0252-0

**Published:** 2015-07-22

**Authors:** Marko Topalovic, Eric Derom, Christian R. Osadnik, Thierry Troosters, Marc Decramer, Wim Janssens, on behalf of the Belgian Pulmonary Function Study Investigators

**Affiliations:** Department of Clinical and Experimental Medicine, Laboratory of Respiratory Diseases, University Hospital Leuven, KULEUVEN University of Leuven, Herestraat 49, 3000 Leuven, Belgium; Respiratory Division, University Hospital Ghent, University of Ghent, Ghent, Belgium; Department of Rehabilitation Sciences, Faculty of Kinesiology and Rehabilitation Sciences, KU Leuven, Leuven, Belgium; Department of Physiotherapy, Monash University, Victoria, Australia; Institute for Breathing and Sleep, Victoria, Australia; Monash Health, Monash Lung and Sleep, Victoria, Australia

**Keywords:** Body-plethysmography, Airway resistance, Pulmonary function tests, Chronic obstructive pulmonary disease, Asthma

## Abstract

**Background:**

Airway resistance (R_AW_) and specific airway conductance (sG_AW_) are measures that reflect the patency of airways. Little is known of the variability of these measures between different lung diseases. This study investigated the contribution of R_AW_ and sG_AW_ to a diagnosis of obstructive airways disease and their role in differentiating asthma from COPD.

**Methods:**

976 subjects admitted for the first time to a pulmonary practice in Belgium were included. Clinical diagnoses were based on complete pulmonary function tests and supported by investigations of physicians’ discretion. 651 subjects had a final diagnosis of obstructive diseases, 168 had another respiratory disease and 157 subjects had no respiratory disease (healthy controls).

**Results:**

R_AW_ and sG_AW_ were significantly different (*p* < 0.0001) between obstructive and other groups. Abnormal R_AW_ and sG_AW_ were found in 39 % and 18 % of the population, respectively, in which 81 % and 90 % had diagnosed airway obstruction. Multiple regression revealed sG_AW_ to be a significant and independent predictor of an obstructive disorder. To differentiate asthma from COPD, R_AW_ was found to be more relevant and statistically significant. In asthma patients with normal FEV_1_/FVC ratio, both R_AW_ and sG_AW_ were more specific than sensitive diagnostic tests in differentiating asthma from healthy subjects.

**Conclusions:**

R_AW_ and sG_AW_ are significant factors that contribute to the diagnosis and differentiation of obstructive airways diseases.

## Background

Whole-body plethysmography is an increasingly frequent and important component of comprehensive pulmonary function testing in respiratory medicine, and an integral diagnostic procedure to evaluate static lung volumes and airway resistance [1, 2]. To determine airways resistance, plethysmography applies the gas law of Boyle-Mariotte [3] to evaluate the difference in the pressure of the closed box (in which the subject is breathing) in conjunction with flows measured at the subject’s mouth by breathing out of the box [4, 5].

The two most commonly used resistive parameters are airway resistance (R_AW_) and specific airway conductance (sG_AW_). R_AW_ reflects changes in alveolar pressure over changes in flow [6] representing true resistance of the airways. R_AW_ is determined by airway narrowing and is therefore considered a good parameter for the diagnosis of airflow obstruction [6]. As the manoeuvre to determine airway resistance is dependent on thoracic gas volume (with larger volumes resulting in opened airways), specific airway resistance (sR_AW_) is divided by thoracic gas volume to obtain R_AW_. Mathematically, sR_AW_ does not represent resistance but the flow-standardized work that one needs to perform in order to complete the manoeuvre [4]. SG_AW_ is the inverse of sR_AW_ and therefore reflects the conductance of the airways independent of lung volumes.

Despite a large body of physiological evidence, clinicians have lost their interest in measures of resistance which are generally perceived as useless in the work-up of clinical problems. A strong case for the use of such measures in clinical practice recently emerged from a large multicentre study in Belgium (the Belgian Pulmonary Function Study). This study demonstrated that regular measurement of airway resistance (both R_AW_ and sG_AW_), one of four most commonly used pulmonary function tests (PFTs), significantly contributes to a reduction in the number of differential diagnoses and accuracy of final diagnosis [7]. Nevertheless, it is not yet clear how such parameters vary between different lung disease groups and the extent to which they may contribute to differentiation between asthma, COPD and healthy subjects. The aims of the study were to a) explore the variability of resistance parameters between established diagnoses of respiratory diseases in a general patient sample, and b) investigate whether measures of resistance can differentiate between the obstructive diseases and further refine the diagnosis of asthma or COPD.

## Methods

### Study subjects

In total 976 subjects were included from the Belgian Pulmonary Function Study. This prospective cohort study includes all successive new subjects from the periods of June 6th till September 12th 2011, and January 16th till June 12th 2012 across 33 hospitals in Belgium. Details on the protocol can be found in the corresponding publication [7]. All subjects were of Caucasian race aged 18 to 75 years with a history of respiratory complaints (dyspnea, cough, sputum, wheezing etc.). All performed complete PFTs at cohort entry including post-bronchodilator spirometry, measurement of diffusion capacity and body-plethysmography including measures of lung volumes, airway resistance (R_AW_) and conductance (sG_AW_, only available in 778 subjects). Pulmonologists performed additional tests such as imaging, ECG and other PFTs to determine diagnosis, where necessary. All final diagnoses for each included subject were validated by local Belgian focus groups of 20 to 25 pulmonologists who jointly evaluated all test outcomes. A final diagnosis was selected from a predefined list of 13 diseases covering the wide spectrum of respiratory diseases that are potentially diagnosed by pulmonary function: asthma, COPD, other obstructive diseases, upper airway obstruction, neuromuscular disease, thoracic or pleural disease, obesity, interstitial lung disease, systemic sclerosis or vascular disease, cardiac failure, hyperventilation, no primary pulmonary abnormality, and others (lung cancer, rhinosinusitis, etc.). The study was registered on clinicaltrials.gov (identifier: NCT01297881).

### Pulmonary function tests

All PFTs were performed according to the ATS/ERS criteria [8] using standardized equipment available in the participating pulmonary function laboratory (including: Sensormedics Whole Body Plethysmograph, Care fusion, Belgium; Masterscreen Jaeger, Care fusion, Belgium; Medisoft boybox 5500, Belgium; Ganshoren-Medisoft Hyp’air compact, Belgium). Spirometry data are post-bronchodilator measures and expressed, together with airway resistance and lung volume measurements as percent predicted of normal reference values [9, 10]. SG_AW_ was calculated by implementing a least-squared fit of the line through the specific resistance loop at a defined fixed flow of ±0.5 L/s [5, 11]. Diffusing capacity (DL,_CO_) was measured by the single-breath carbon monoxide gas transfer method and expressed as percent predicted of reference values [12].

Initial inspection of resistance values (expressed as % predicted) from healthy subjects (no primary pulmonary disease) revealed slightly different resistance values than anticipated. This may be due to limited setting where existing reference values for resistive parameters were developed. We therefore established new upper and lower limits of normality for R_AW_ and sG_AW_ based on a 90 % reference interval from our large healthy reference population (*N* = 157). The new proposed upper limit of normality for R_AW_ was 0.38 (kPa/L/s), corresponding to 173 % of the median healthy population reference value. The new lower limit of normality for sG_AW_ was 0.63 (1/kPa*sec) corresponding to 74 % of the reference value. As with existing reference equations on R_AW_ (median value 0.22) and sG_AW_ (median value 0.85), no gender separation was made as no influence on median resistive values of our healthy reference population was observed.

To explore independency of R_AW_ and sG_AW_ on an individual patient level, we compared it with other lung function parameters indicative of the presence of airflow obstruction. These included: FEV_1_/FVC ratio, MMEF, RV, TLC and FRC. If one of the latter was out of the predicted normal range, it was considered to be disturbed. Increases in the number of disturbed measures were associated with increased probability of the presence of ‘true’ airflow obstruction.

### Statistical analysis

Statistical Analysis System (SAS) version 9.3, (SAS Institute, Cary, USA) was used for all statistical analyses. A Shapiro-Wilk test was used to explore data normality of each group, and differences between two groups were analysed via unpaired t-tests or Mann–Whitney tests where normal or non-normal data distribution existed, respectively. Logistic-regression models were applied for analysis of binary variables using stepwise selection to identify the subset of variables with the strongest relation to the presence of obstruction or specific disease diagnoses. The default criterion for variables entering or egressing the models was significance at the 0.15 level. Receiver operating characteristic (ROC) curve analysis was performed via GraphPad Prism version 5.01, (GraphPad Software, La Jolla, California, USA) to identify the best cut-off values of R_AW_ and sG_AW_ to distinguish between asthma and COPD diagnoses. New reference values were determined using MedCalc 14.8.1 (MedCalc Software bvba, Ostend, Belgium), with Tukey test applied to detect outliers.

## Results

### Baseline characteristics

Baseline characteristics of all included subjects are summarised in Table 1 according to three groups, based upon final diagnosis. The ‘healthy’ group included subjects without primary pulmonary abnormality; the ‘obstruction’ group included subjects with COPD, asthma, upper airway obstruction and other obstructive diseases (e.g. chronic bronchitis, bronchiolitis, small airways disease, bronchiectasis, cystic fibrosis); and the ‘other pulmonary diseases’ group comprised subjects with other, non-obstructive pulmonary diseases. Consistent with clinical practice, the ‘obstruction’ group was the most common diagnostic group (*N* = 651, 67 % of total sample), followed by ‘other pulmonary diseases’ (*N* = 168, 17 %) and ‘healthy’ (*N* = 157, 16 %).

**Table 1:** Study population characteristics

	Healthy	Obstruction	Other pulmonary diseases
Subjects, n	157	651	168
Age, years	53 (41–64)	55 (42–65)	57 (44–67)
Smoking, pack yr.	0 (0–5)	0 (0–30)	0 (0–15)
BMI, kg/m^2^	25.3 (21.6–28.7)	25.2 (21.1–29.7)	27.6 (23.1–32.0)
FEV_1_, %predicted	103.3 (91.5–114.7)	84.2 (67.0–97.7)	88.1 (74.4–103.8)
FVC, %predicted	106.6 (97.2–114.9)	97.7 (84.4–110.4)	92.1 (76.5–107.8)
DL_CO_, %predicted	86.4 (77.0–99.2)	78.7 (63.0–89.9)	70.3 (56.3–85.7)
RV, %predicted	106.9 (91.6–129.7)	131.6 (104.0–159.9)	101.8 (80.9–124.6)
TLC, %predicted	104.6 (95.7–112.1)	106.0 (94.8–117.1)	88.6 (79.7–103.4)
FRC, %predicted	105.1 (93.4–123.1)	119.4 (99.2–143.3)	95.7 (78.0–116.5)
R_AW_, kPa/L/s	0.24 (0.18–0.30)	0.32 (0.23–0.45)	0.25 (0.17–0.35)
sG_AW_, 1/ kPa*sec	1.21 (0.95–1.57)	0.84 (0.56–1.17)	1.28 (0.90–1.71)

Definition of abbreviations: *BMI* body mass index, *D*_*L,CO*_ carbon monoxide diffusing capacity, *FEV*_*1*_ forced expiratory volume in one second, *FRC* functional residual capacity, *FVC* forced vital capacity, *RV* residual volume, *R*_*AW*_ airway resistance, *sG*_*AW*_ specific airway conductance, *TLC* total lung capacity, Values are median and IQR

### Value of resistance parameters for diagnosis of obstructive airways disease

**Fig. 1:**
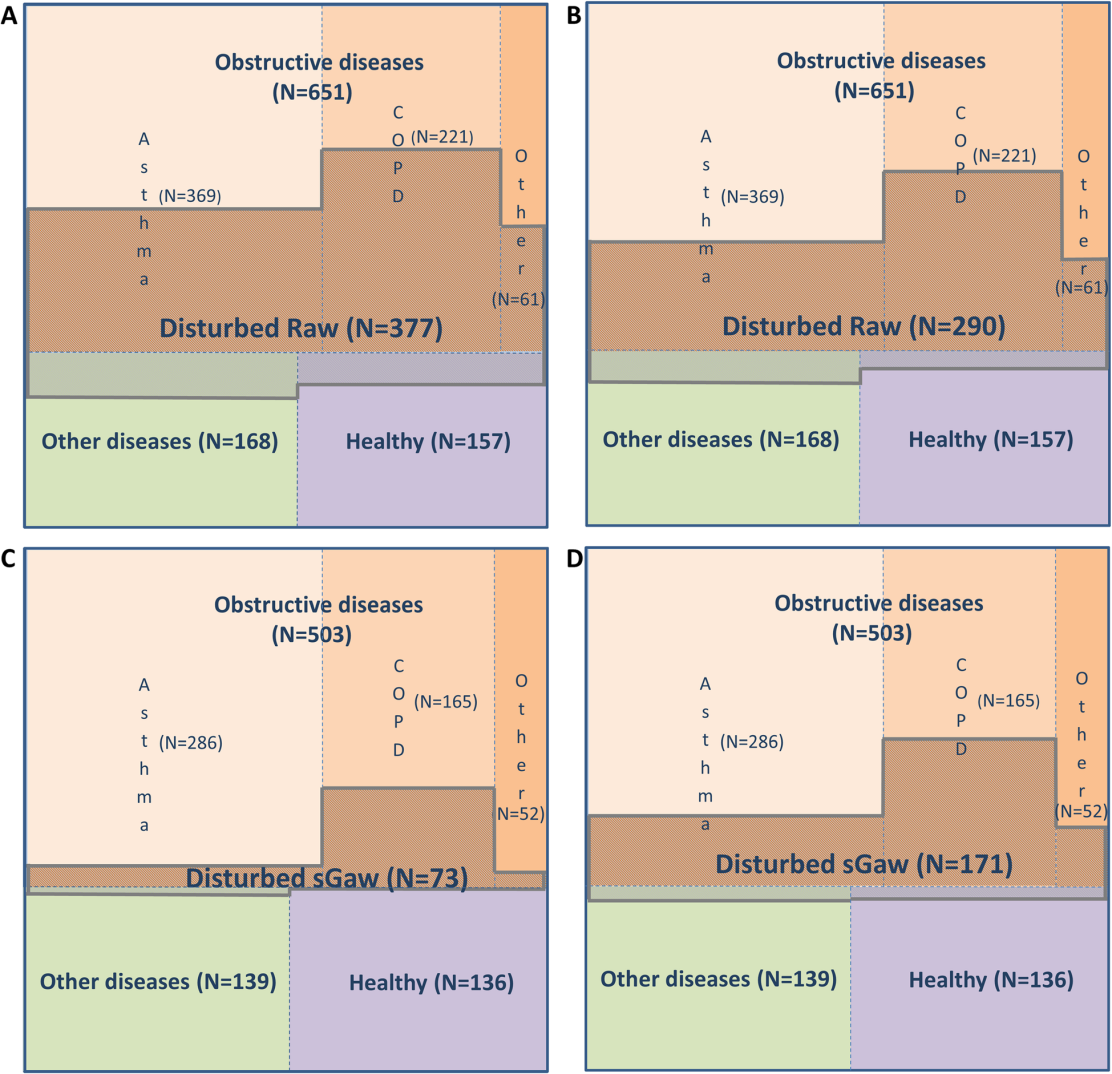
Comparison of R_AW_ and sG_AW_ disturbance within each group of diseases and specific obstructive disease. Each panel is divided into three sections indicating group of diseases and level of disturbance of parameters within each group. Obstructive group is additionally divided into three subsections representing specific obstructive diseases. Panels **a** and **c**/ R_AW_ and sG_AW_ using standard normality limit for disturbance (>150 %, < 50 % of predicted values); Panels **b** and **d**/ R_AW_ and sG_AW_ using statistically drawn limits of healthy subgroup for disturbance; Disturbance is indicated with the cross hatch in the figure; Data from *N* = 976 (R_AW_) and *N* = 778 (sG_AW_) subjects, respectively

Median R_AW_ values were greater and median sG_AW_ values lower in the group of obstructive patients compared to both the healthy controls and the group of non-obstructive respiratory diseases (Table 1). These differences were highly statistically significant (both *p* < 0.0001). Expressed as a percentage of predicted values using the reference equation of Quanjer [10], the total study cohort proportion of disturbed R_AW_ (defined as >150 % predicted of the fixed median value) was 39 % (*N* = 377), whilst for sG_AW_ (defined as <50 % predicted) it was 9 % (*N* = 73). Eighteen percent of healthy controls had an abnormal R_AW_ and one had an abnormal sG_AW_. Implementation of the lower and upper limits of normal for R_AW_ and sG_AW_ developed from our own healthy population resulted in a significant change in the proportion of detected abnormalities. Increased R_AW_ (0.38 kPa/L/s; >173 % predicted) was observed in only 30 % (*N* = 290) of subjects, whilst reduced sG_AW_ (0.63 1/kPa*sec; <74 % predicted) was observed in 22 % (*N* = 171) of subjects (Fig. 1). Independently of the cutoffs used, the group of patients with obstructive diseases had a significant higher proportion of increased R_AW_. In particular, 47 % of all subjects with obstructive airways disease (Panel A) or 37 % if using the less stringent cut-off (Panel B), presented with an increased resistance. A reduced sG_AW_ (Panels C and D) had a high positive predictive value for obstructive diseases, as 92 % or 90 % (depending of cut-off) of all cases with low sG_AW_ presented with such a diagnosis. This was confirmed in the multiple logistic regression analysis, which identified sG_AW_ as a significant contributing factor to a diagnosis of obstructive lung disease (Table 2). To verify whether resistance parameters (expressed as percent predicted) are beneficial and additional to other PFT parameters to diagnose obstructive airflow disorders, we created different regression models comprising parameters of spirometry only, parameters from spirometry and body-plethysmography and by the combination of the former including diffusing capacity. Stepwise selection excluded R_AW_ in all models, however sG_AW_ contributed significantly to all lung function models.

**Table 2:** Relationship between pulmonary function parameters and the presence of obstruction in multiple logistic regression with stepwise selection

Variables	Odds Ratio (95 % Confidence limit)	p value
1.		
sG_AW_, %predicted	0.996 (0.994–0.998)	0.0003
MEF75, %predicted	0.980 (0.972–0.988)	<0.0001
FEV_1_/FVC	0.936 (0.912–0.960)	<0.0001
2.		
TLC, %predicted	1.058 (1.043–1.075)	<.0001
sG_AW_, %predicted	0.997 (0.995–0.999)	0.0031
MMEF, %predicted	0.987 (0.978–0.996)	0.0108
MEF75, %predicted	0.979 (0.969–0.988)	<.0001
FEV_1_, %predicted	0.970 (0.955–0.986)	<.0001

1/ Variables of spirometry and resistance (initial model1: FVC, %pred, FEV_1_, %pred, FEV_1_/FVC, MEF75, %pred, MEF50, %pred, MEF25, %pred, MMEF, %pred, PEF, %pred. R_AW_, %pred. and sG_AW_, %pred.); 2/ added variables of lung volumes (initial model2: model1, TLC, %pred, RV, %pred, FRC, %pred.); Adding variables of diffusion capacity (initial model3: model2, DL_CO_, %pred, K_CO_, %pred.) resulted in same values as model2

The presence of abnormal R_AW_ and sG_AW_ values rarely occurred in isolation and was commonly observed in conjunction with disturbances of other PFT parameters indicative of obstruction (Table 3). This was particularly evident for sG_AW_ where only few subjects had normal values of all other PFT parameters. Of the nine cases of isolated sG_AW_ disturbance, two were diagnosed as healthy, one had neuromuscular disease, one had another obstructive disease, and five had asthma. Additionally, most subjects who presented with isolated abnormally high R_AW_ values had a diagnosis of asthma with normal lung function. Application of the classical cut-offs resulted in detection of 54 subjects with disturbed resistance, of which 28 had asthma, 11 were considered healthy, one had COPD, five presented with other obstructive diseases and nine had various other respiratory diseases. Our more restrictive cut-off threshold identified 29 instances of disturbed resistance, of which 18 had asthma, six were considered healthy and five had other respiratory diseases.

**Table 3:** Disturbed resistance in relation with the disturbance of other lung function parameters indicative of obstruction

R_AW_ (>150 %pred.)		R_AW_ (>173 %pred.)		sG_AW_ (<50 %pred.)		sG_AW_ (<74 %pred.)	
Alone	Together	Alone	Together	Alone	Together	Alone	Together
54 (=20 %)	218	29 (=14 %)	176	1 (=1.5 %)	68	9 (=6 %)	147

Alone: disturbance of only R_AW_ or sG_AW_. Some patients excluded from analysis due to missing measurement data. Included parameters: FEV_1_/FVC, MMEF, RV, TLC and FRC

### Value of resistance parameters for differentiating asthma from COPD in obstructive airways diseases

Due to the lack of available data for sG_AW_ in some subjects, diagnostic groups differed in size from the principal analysis, but not in their proportion relative to each other. Median (IQR) R_AW_ values for the asthma (*N* = 369) and COPD (*N* = 221) groups were 0.30 kPa/L/s (0.22–0.42) and 0.39 kPa/L/s (0.26–0.53), respectively (*p* < 0.0001). For sG_AW_ (*N* = 286 for asthma; *N* = 165 for COPD), these were 0.90 1/kPa*sec (0.69–1.22) and 0.60 1/kPa*sec (0.41–0.98), respectively (*p* < 0.0001).

When using the complete set of PFTs in the group of patients with obstructive diseases, application of multiple logarithmic regression with stepwise selection did not reveal an independent statistical role for R_AW_ or sG_AW_ in the differentiation between asthma and COPD. Although R_AW_ was statistically significant in models involving spirometry and plethysmography parameters only, the better variable of differentiation was diffusion capacity (Table 4). When looking at the cumulative disturbances of other PFT parameters per diseased subject, in COPD, the presence of disturbed R_AW_ or sG_AW_ was associated with abnormalities in several other PFT parameters. For those with asthma, increased R_AW_ more commonly occurred in isolation (Table 5). The latter may suggest a more specific role of R_AW_ in the diagnosis of asthma with a non-obstructive spirometry.

**Table 4:** Relationship between pulmonary function parameters and the presence of asthma in multiple logistic regression with stepwise selection (subjects with obstructive diseases only)

Variables	Odds Ratio (95 % Confidence limit)	p value
1.		
FEV_1_/FVC	1.043 (1.012–1.074)	<0.0001
MEF25, %predicted	1.024 (1.010–1.038)	0.0024
PEF, %predicted	1.018 (1.006–1.030)	0.0088
R_AW_, %predicted	1.002 (1.000–1.004)	0.0320
2.		
FEV_1_, %predicted	1.029 (1.013–1.046)	<.0001
MEF25, %predicted	1.022 (1.010–1.035)	0.0015
R_AW_, %predicted	1.002 (1.000–1.004)	0.0287
FRC, %predicted	0.981 (0.973–0.989)	<.0001
3.		
DL_CO_, %predicted	1.071 (1.051–1.090)	<.0001
FEV_1_/FVC	1.051 (1.024–1.078)	<.0001
TLC, %predicted	1.040 (1.014–1.067)	0.0021
FRC, %predicted	0.970 (0.956–0.984)	0.0041

1/ Variables of spirometry and resistance (initial model1: FVC, %pred, FEV_1_, %pred, FEV_1_/FVC, MEF75, %pred, MEF50, %pred, MEF25, %pred, MMEF, %pred, PEF, %pred. R_AW_, %pred. and sG_AW_, %pred.); 2/ added variables of lung volumes (initial model2: model1, TLC, %pred, RV, %pred, FRC, %pred.); 3/ added variables of diffusion capacity (initial model3: model2,DL_CO_, %pred, K_CO_, %pred.)

**Table 5:** Number of subjects with disturbance per disease (when resistance or specific conductance is already disturbed) stratified over total number of disturbed lung function parameters indicative of obstruction

Disturbance	R_AW_ (>150 %pred.) *N* = 204 (=31 %)		R_AW_ (>173 %pred.) *N* = 162 (=25 %)		sG_AW_ (<50 %pred.) *N* = 61 (=12 %)		sG_AW_ (<74 %pred.) *N =* 133 (=26 %)	
	Asthma (*N =* 112)	COPD (*N =* 92)	Asthma (*N =* 85)	COPD (*N =* 77)	Asthma (*N =* 16)	COPD (*N =* 45)	Asthma (*N =* 55)	COPD (*N =* 78)
0	28	1	18	0	0	0	5	0
1	27	7	23	6	4	0	12	3
2	25	12	19	11	2	2	12	8
3	20	11	16	7	5	4	16	7
4	9	32	6	26	4	19	9	29
5	3	29	3	27	1	20	1	31

Some patients excluded from analysis due to missing measurement data. Proportions observed in a group of all subjects with obstructive diseases. Included parameters: FEV_1_/FVC, MMEF, RV, TLC and FRC

### Resistance and specific conductance in non-obstructive asthma

In COPD, R_AW_ and sG_AW_ were never independently disturbed as significant changes always concurred with significant changes of other PFT measures, including the FEV_1_/FVC ratio by definition. In contrast, subjects with asthma, 28 of 112 (28 %) or 18 of 85 (21 %) presented with isolated R_AW_ disturbance. To further examine this independent value of resistance in asthma, we focussed on the subgroup of asthmatics and healthy controls with normal FEV_1_/FVC ratio (above LLN) which resulted in a group of 285 subjects with available R_AW_ data and 213 subjects with available sG_AW_ data. When applying the maximal sum of sensitivity and specificity to select the best R_AW_ cut-off for asthma diagnosis in that particular subgroup, ROC curve identified an ideal cut-off value of 0.31 kPa/L/s, with a specificity of 76 %, sensitivity of 45 % and positive predictive value of 78 % (Table 6, panel A). For sG_AW_ a cut-off value of 0.98 was identified with associated specificity of 74 %, sensitivity of 50 % and positive predictive value of 75 % (Table 6, panel B).

**Table 6:** Value of resistance to differentiate between healthy subjects and asthma patients with normal FEV_1_/FVC ratio

		Asthma (N)	Control (N)	Total (N)
A	R_AW_ > 0.31	129	37	166
	R_AW_ ≤ 0.31	156	120	276
	Total (N)	285	157	442
B	sG_AW_ < 0.98	106	35	141
	sG_AW_ ≥ 0.98	107	101	208
	Total (N)	213	136	349

## Discussion

Our study demonstrates measures of airway resistance and specific conductance have a valuable and potentially important role in the diagnosis of obstructive diseases. Specific conductance clearly contributes to diagnosis of obstructive airways diseases, whilst airway resistance can help differentiate between asthma and COPD. The presence of abnormal resistive parameters in isolation from other PFT measures may help differentiate between asthma with normal FEV_1_/FVC ratio and normal, good health.

Questions regarding the relevance of resistive parameters for diagnosing respiratory diseases are certainly not new. Prior to this study, it has been shown that specific resistance (reciprocal of specific conductance) may indicate bronchial reversibility in asthma, as it has a different physiological meaning than FEV_1_ [13]. Most of its contribution, however, is demonstrated in children with asthma where measurement of specific resistance has been shown to improve diagnostic accuracy and decrease diagnostic delay [14–16]. Interestingly, it has also been shown that specific resistance has a better role in predicting asthma then airway resistance [17], and has higher sensitivity than FEV_1_ in terms of detecting lung function changes during methacholine challenges [18]. In COPD, both specific conductance and airway resistance are more sensitive for assessing short-acting bronchodilator effects than FEV_1_ [18, 19]. Increased airway resistance and decreased specific conductance have also been detected as early features of mild COPD [20, 21]. Detailed analysis of computer tomography scans shows that increased airway resistance in COPD is correlated with the thickening of the airway walls [22]. Our data are in line with these earlier observations, but provide a more comprehensive overview of the role of resistance and specific conductance in a large unbiased sample of a routine respiratory practice in conjunction with other pulmonary function tests. Although resistance and specific conductance overlap with the disturbed pattern of pulmonary function tests in patients with a respiratory disorder, in some subtypes, they may reveal unique components to suggest a certain diagnosis. We demonstrate that both parameters have their specific characteristics which are not the leading but yet still significant contributors to respiratory disease diagnosis.

As expected, the highest frequency of abnormal resistance and specific conductance was found in patients with obstructive airways diseases, particularly COPD. R_AW_ abnormalities were present in 50 % of subjects with mild COPD stages (former GOLD I and II, 75 % of all COPD cases), This proportion rose to 91 % in those with more severe disease (former GOLD III and IV). Whilst fewer subjects had disturbed sG_AW_, specific conductance appeared more useful than resistance in distinguishing obstructive diseases from non-obstructive, healthy or restrictive diseases. To some extent this may be explained by the fact that sG_AW_ is independent of the thoracic gas volume [23] and may thus differentiate disturbed resistances of smaller airways at low lung volumes (e.g. interstitial lung disease) from real intraluminal narrowing or collapse, in cases of asthma and COPD [24]. The observation that R_AW_ pops up again when it comes to the further differentiation of asthma from COPD, may be explained by the fact that disproportionally low resistances (by high thoracic gas volumes) for the level of airflow limitation and FEV_1_ reduction, is a specific characteristic of COPD [25]. Moreover, our data demonstrate that an isolated increase of R_AW_ might be associated with a diagnosis of asthma when airflow limitation on spirometry is not present. Mechanistically, disturbed FEV_1_ measures may be seen as a consequence of increased airway resistance from central and peripheral airways. However, FEV_1_ is obtained from the first second of forced expiration and therefore poorly representative of the small airways. The latter are better visualized at the end of a forced expiration or eventually, by direct measures of resistance during tidal breathing [26, 27].

The most appropriate predictive cut-off values to identify normality of resistive and conductive parameters are contentious. A thorough evaluation of our healthy subject population using the current recommended reference equations yielded unsatisfactory results and indicated that the current reference values may not be up to date [10, 28]. Evaluation of these data using the predicted median and 90 % confident intervals in our healthy reference population (which was considered healthy after an extensive evaluation including all necessary tests) showed the cut-offs for R_AW_ to be too liberal and the cut-offs for sG_AW_ too restrictive. Despite calls for revision of these reference equations from different authors, no official action has been taken yet [28, 29]. Solving this problem may lead to a more accurate and better usability of this test in clinical settings as current predictive values were not constituted in a large and healthy representative population.

An inherent limitation of our multi-centre study may lie in its design to measure pulmonary function using variety of equipment, since it can affect variability and consistency of obtained measurements. Weaknesses of airway resistance and specific conductance are also apparent within the analysis. For example, it is clear that both resistive parameters have large variability within core groups, making them weak independent factors for disease differentiation [30]. Another weakness lies in the estimation process for specific conductance, which affects estimation of airway resistance as well. Practically, the line fitted through the resistance loop to calculate sG_AW_ does not always accurately reflect the entire loop pattern. Often, these differences can be visually detected (e.g. widening of the loop, club-shaped format, S pattern, etc. [4]) but may result in similar inclinations. Additional exploration specifically focused on the pattern of such resistive loops will increase the value of these parameters and may result in a better description of certain phenotypes.

Taken together, airway resistance and specific conductance are certainly not the grand slam winning parameters of pulmonary function tests, however they can significantly contribute to the diagnosis of obstructive diseases and further disease differentiation. Our data also indicate that changing the upper limit of normality for R_AW_ to 0.38 kPa/L/s and accepting a new lower limit of normality for sG_AW_ (0.63 1/kPa*sec) may even improve their diagnostic value.
